# Stress Induced Hyperglycemia and the Subsequent Risk of Type 2 Diabetes in Survivors of Critical Illness

**DOI:** 10.1371/journal.pone.0165923

**Published:** 2016-11-08

**Authors:** Mark P. Plummer, Mark E. Finnis, Liza K. Phillips, Palash Kar, Shailesh Bihari, Vishwanath Biradar, Stewart Moodie, Michael Horowitz, Jonathan E. Shaw, Adam M. Deane

**Affiliations:** 1 Department of Critical Care Services, Royal Adelaide Hospital, Adelaide, South Australia, Australia; 2 Discipline of Acute Care Medicine, University of Adelaide, Level 5 Eleanor Harrald Building, Adelaide, South Australia, Australia; 3 Discipline of Medicine, University of Adelaide, Level 6 Eleanor Harrald Building, Adelaide, South Australia, Australia; 4 Department of Endocrinology, Royal Adelaide Hospital, Adelaide, South Australia, Australia; 5 Department of Critical Care Medicine, Flinders University, Bedford Park, South Australia, Australia; 6 Department of Intensive Care Medicine, Flinders Medical Centre, Bedford Park, South Australia, Australia; 7 Department of Intensive Care Medicine, Lyell McEwin Hospital, Elizabeth Vale, South Australia, Australia; 8 Baker IDI Heart and Diabetes Institute, Melbourne, Victoria, Australia; Baylor College of Medicine, UNITED STATES

## Abstract

**Objective:**

Stress induced hyperglycemia occurs in critically ill patients who have normal glucose tolerance following resolution of their acute illness. The objective was to evaluate the association between stress induced hyperglycemia and incident diabetes in survivors of critical illness.

**Design:**

Retrospective cohort study.

**Setting:**

All adult patients surviving admission to a public hospital intensive care unit (ICU) in South Australia between 2004 and 2011.

**Patients:**

Stress induced hyperglycemia was defined as a blood glucose ≥ 11.1 mmol/L (200 mg/dL) within 24 hours of ICU admission. Prevalent diabetes was identified through ICD-10 coding or prior registration with the Australian National Diabetes Service Scheme (NDSS). Incident diabetes was identified as NDSS registration beyond 30 days after hospital discharge until July 2015. The predicted risk of developing diabetes was described as sub-hazard ratios using competing risk regression. Survival was assessed using Cox proportional hazards regression.

**Main Results:**

Stress induced hyperglycemia was identified in 2,883 (17%) of 17,074 patients without diabetes. The incidence of type 2 diabetes following critical illness was 4.8% (821 of 17,074). The risk of diabetes in patients with stress induced hyperglycemia was approximately double that of those without (HR 1.91 (95% CI 1.62, 2.26), *p*<0.001) and was sustained regardless of age or severity of illness.

**Conclusions:**

Stress induced hyperglycemia identifies patients at subsequent risk of incident diabetes.

## Introduction

Stress induced hyperglycemia occurs in critically ill patients in whom glucose tolerance was previously normal, with hyperglycemia resolving following recovery [1]. This acute derangement is observed frequently; within 48 hours of intensive care unit (ICU) admission up to 50% of critically ill patients are hyperglycemic [2]. Stress induced hyperglycemia is known to be a marker of illness severity, with the magnitude of hyperglycemia strongly associated with short-term mortality, particularly in patients without a history of diabetes [2, 3].

The pathophysiology of stress induced hyperglycemia is thought to reflect temporary insulin resistance coupled with relative insulin deficiency, in that plasma insulin concentrations are inadequate to compensate for hyperglycemia [1]. Insulin resistance is driven by the stress response to critical illness initiating an overwhelming activation of pro-inflammatory mediators (tumour necrosis factor-α, interleukin-6) and counter-regulatory hormone excess (glucagon, cortisol, catecholamines) which lead to excessive hepatic gluconeogenesis and down-regulation of insulin-mediated GLUT-4 glucose transporters [4]. Whether critical illness unmasks latent insulin resistance and/or impaired β-cell function has not been adequately explored. The identification of long-term metabolic derangements that are amenable to intervention is important, particularly because outcomes for survivors of critical illness remain poor, with up to 40% of patients dying within five years of hospital discharge [5].

The concept that transient hyperglycemia during critical illness identifies patients at increased risk for developing type 2 diabetes is intuitively plausible. For example, there are similarities between critical illness and gestational diabetes where an acute period of glucose intolerance initially normalises following resolution of the physiological challenge [6, 7]. While gestational diabetes was once considered a temporary disorder of pregnancy, it is now recognised that affected women are at high risk of developing type 2 diabetes [8, 9]. Moreover, screening programs are advocated to detect early impairment in glucose tolerance because intervention strategies in at-risk populations have been shown to reduce the progression to type 2 diabetes [9, 10].

The primary aim was to evaluate the association between peak blood glucose in the first 24 hours of ICU admission and the subsequent risk of incident diabetes in survivors of critical illness.

## Research Design and Methods

The protocol was approved by the Research Ethics Committee of the Royal Adelaide Hospital, the South Australian Department of Health and the Australian Institute of Health and Welfare, with the need for informed consent waived. Access to data for the purpose of performing this research was approved by the National Diabetes Service Scheme, maintained by Diabetes Australia, and by the South Australian Department of Health with third-party data matching approved by the Australian Institute of Health and Welfare.

### Patients

This was a retrospective, multi-centre observational study across all public hospital ICUs in South Australia. Public intensive care services in South Australia (population 1.7 million) are exclusively provided by four tertiary hospitals (Flinders Medical Centre, Lyell McEwin Hospital, Queen Elizabeth Hospital and Royal Adelaide Hospital). Patient demographic, hospital episode and intensive care admission data were extracted from each contributing ICU from January 1 2004 to December 31 2011 inclusive. At each unit these data were collected prospectively prior to submission to the Australia and New Zealand Intensive Care Society Adult Patient Database (ANZICS-APD) [11]. The ANZICS-APD captures clinical, physiological, and laboratory data for the initial 24 hours of ICU admission, along with outcome data, for all patients admitted to ICUs across Australia and New Zealand [12]. These data were then linked to population based datasets to match (i) International Classification of Diseases (ICD-10) coding of diabetes through the Department of Health Integrated South Australian Activity Collection dataset, generating a “known diabetes” flag for each hospital separation [13], (ii) socio-economic status, estimated from the separation postcode using the Australian Bureau of Statistics Index of Relative Socio-Economic Advantage and Disadvantage [14], (iii) mortality, through the Australian National Death Index and (iv) incident diabetes, through registration with the Australian National Diabetes Service Scheme (NDSS). The Australian Institute of Health and Welfare performed data linkage between the composite ICU dataset and the NDSS dataset and National Death Index. The NDSS dataset has more than one million Australians registered as having type 2 diabetes [15].

Patients over the age of 18 years who survived ICU and were discharged from hospital alive were assigned to one of three groups; (i) prevalent or ‘known’ diabetes, where either (a) ICD-10 codes from the diabetes chapter (E10-E14) were present in the current or any prior hospital separation, either as a principal diagnosis or a complication, (b) the patient was registered with the NDSS as having diabetes prior to, or within 30 days of hospital separation, or (c) the peak blood glucose was > 20 mmol/L (360 mg/dL) [16], (ii) stress induced hyperglycemia (SIH), where diabetes was not prevalent (defined as above) and a recorded blood glucose ≥ 11.1 mmol/L (200 mg/dL) [2], and (iii) the control group, where diabetes was not prevalent and all blood glucose levels were < 11.1 mmol/L (200 mg/dL). For patients with multiple ICU and/or hospital episodes during the study period only the index admission was used such that each patient was only included once in the final analysis.

New registration with the NDSS was used as a surrogate measure for incident diabetes, with time to registration from 30 days post hospital discharge forming the primary study outcome, as per McAllister and colleagues [16]. Secondary outcomes included the assessment of covariates potentially influencing the time to NDSS registration and description of the survival patterns between those with and without stress induced hyperglycemia.

### Statistical analysis

Data are presented as frequencies and proportions for categorical variables and mean (standard deviation) or median [interquartile range] for continuous variables. For NDSS registration, time to event analysis was described as sub-hazard ratios using competing risk regression, based upon the approach of Fine and Gray [17]. This was planned *a priori*, as death could not be considered a ‘non-informative’ censoring event. A sensitivity analysis was performed using Cox proportional hazards regression, treating death as a censoring event. Patient survival was assessed using Cox proportional hazards regression. For competing risks and Cox proportional hazards models, between group effects are presented as sub-hazard ratio, SHR (95% CI) and hazard ratio, HR (95% CI) respectively. Peak blood glucose levels between participating units were compared using linear regression with ‘hospital’ as an indicator variable. Between group comparisons were considered statistically significant at *p* < 0.05. Inclusion of covariates in multivariate models was set at *p* < 0.1. All analyses were performed using Stata/MP 14.1 software.

## Results

### Baseline characteristics

**Fig 1:**
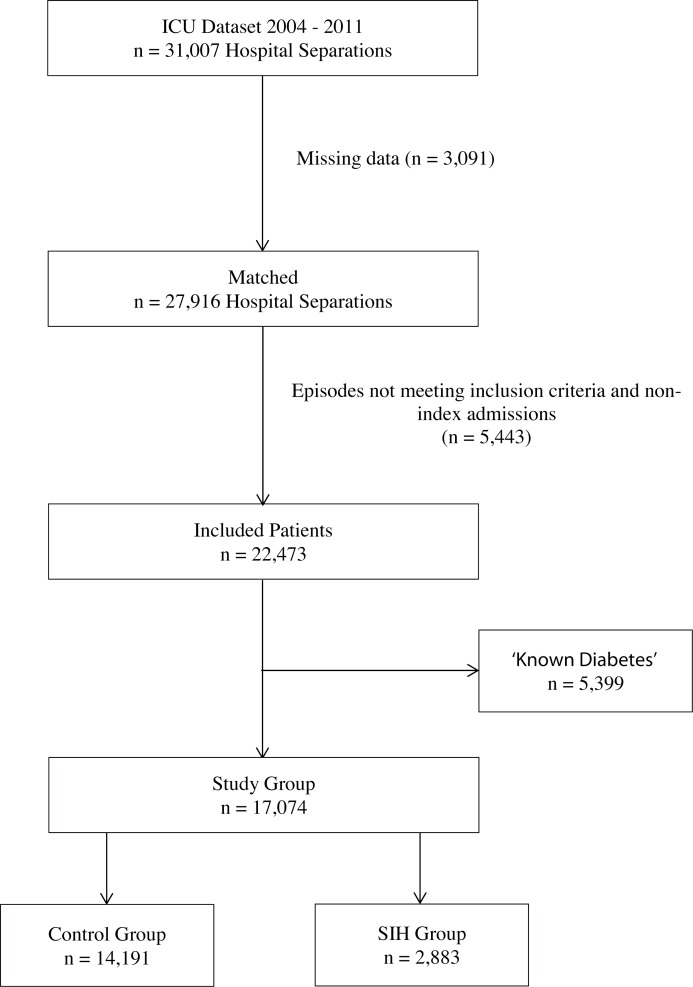
CONSORT style flowchart of patients included in analysis. SIH, stress induced hyperglycemia.

A total of 31,007 patient separations were recorded during the capture period; 3,091 of these had missing data, 1,292 with no matching SA Health record and 1,799 no available blood glucose result. A further 5,443 were excluded due to either non-index ICU or hospital admission status (5,078) or age less than 18 years (365), leaving 22,473 index separations. There were 5,399 (24%) patients with prevalent diabetes, leaving 17,074 patients for analysis; of these, 2,883 (17%) fulfilled the criteria for stress induced hyperglycemia and 14,191 (83%) formed the control group (Fig 1). Demographic data are presented in Table 1. Patients were followed-up for a maximum of 8 years post-discharge, with a median follow-up of 5.3 [3.6, 7.5] years.

**Table 1:** Demographic data by study group.

	Normoglycemia	Stress Induced Hyperglycemia	Total
Separations, n (% total)	14,191 (63)	2,883 (13)	17,074
Male, n (% group)	8,522 (60)	1,635 (57)	10,157 (59)
Site, n (% group)			
A	5,009 (35)	961 (33)	5,970 (35)
B	5,180 (37)	996 (35)	6,176 (36)
C	2,245 (16)	576 (20)	2,821 (17)
D	1,757 (12)	350 (12)	2,107 (12)
ATSI, n (% group)	443 (3.1)	72 (2.5)	515 (3.0)
Age, mean (SD)	56.7 (20.0)	61.8 (17.6)	57.4 (19.7)
APACHE III, med (IQR)	52 (36, 69)	67 (50, 87)	54 (38, 72)
Length of Stay, med (IQR)			
ICU	1.9 (1.0, 3.9)	2.7 (1.4, 5.6)	2.0 (1.0, 4.1)
Hospital	11.1 (5.9, 21.1)	13.0 (7.2, 24.6)	11.4 (6.1, 21.7)
Acute Renal Failure, n (% grp)	308 (2.2)	111 (3.9)	419 (2.5)
Peak BG, med (IQR)	7.8 (6.6, 9.0)	12.7 (11.7, 14.4)	8.3 (6.9, 10)
Medical, n (% group)	8,463 (59.6)	1,910 (66.3)	10,373 (60.8)
Surgical, n (% group)	5,728 (40.4)	973 (33.8)	6,701 (39.3)
Trauma, n(% group)	1,552 (10.94)	165 (5.72)	1,717 (10.6)

ATSI Aboriginal and Torres Strait Islander peoples, APACHE Acute Physiology and Chronic Health Evaluation, BG Blood Glucose (mmol/L).

### Peak blood glucose differences between participating units

Despite minor heterogeneity in the glucose threshold at which intravenous insulin was commenced at each site (S1 Table), the mean peak glucose concentrations differed by 0.6 mmol/l (10.8 mg/dL) between participating units (range 8.5–9.1 mmol/l (153–164 mg/dL), *P* < 0.0001, S1 Fig).

### NDSS capture rate of patients with prevalent diabetes

Of the 5,399 patients with prevalent diabetes, 4,176 were diagnosed based on ICD-10 coding within the SA Health dataset. Following matching with the NDSS, 3,363 of these patients were registered with the National Diabetes Service Scheme, reflecting a capture rate of 80.5%.

### Risk of incident diabetes post ICU discharge

**Fig 2:**
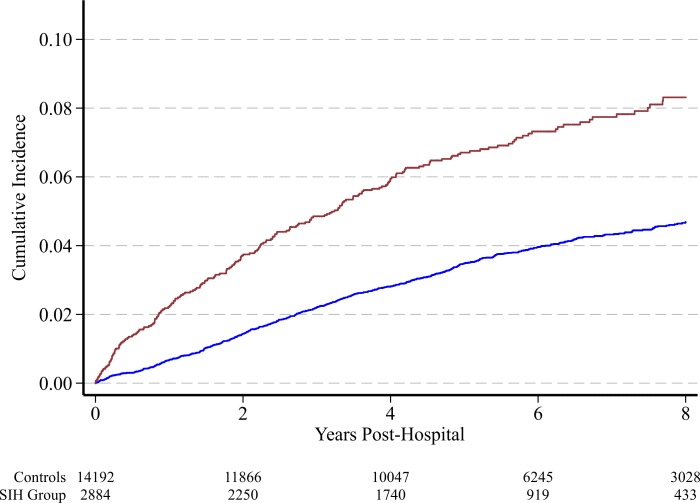
Cumulative incidence for type 2 diabetes for the control group (blue line) versus stress induced hyperglycemia (red line).

Within the follow-up period, 4.8% (821 of 17,074) of patients newly registered with the NDSS as having diabetes. Stress induced hyperglycemia increased the risk of incident diabetes with a relative sub-hazard ratio 1.88 (1.61, 2.20), *P* < 0.001 (Fig 2). Demographic covariates (Table 1) were entered into a multivariate model, along with an age-squared term given observed non-linearity. Backwards elimination was employed, retaining covariates significant at P < 0.10; this included age, age-squared, severity of illness (APACHE III), hospital, socioeconomic status, acute renal failure, medical diagnosis and trauma. After adjustment, stress induced hyperglycemia remained an independent risk factor for incident diabetes, with an adjusted sub-hazard ratio of 1.91 (1.62, 2.26), *P* < 0.001. A post hoc sensitivity analysis limiting the duration of observation to four years did not alter this signal (adjusted sub-hazard ratio: 2.16 (1.8, 2.58)). There was a positive non-linear relationship between NDSS registration and maximum blood glucose level, with a marked increase in risk at blood glucose levels >7.8 mmol/L (S2 Fig). A sensitivity analysis performed against SIH, with a cut-off blood glucose value of 7.8 mmol/L [18, 19], was associated with a sub-hazard ratio of 2.07 (1.78, 2.43), *P* <0.0001).

### Effect of age on risk of incident diabetes

**Fig 3:**
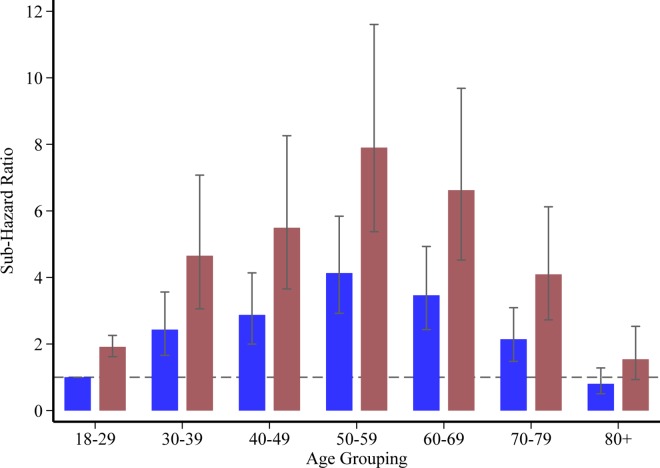
Sub-hazard ratios for the risk of type 2 diabetes by glycemic category and age group. Control group = blue bars; stress induced hyperglycemia = red bars. Age is grouped approximately into deciles with normoglycemia and age 18–29 years as the base reference; Data are sub-hazard ratios ± 95% confidence intervals.

The association of age with the risk of incident diabetes was non-linear, with both age and age-squared terms being significant in the multivariate model, P<0.0001 respectively. In order to visualise this relationship, age was grouped into approximate deciles and the risk of diabetes estimated for each group; peak risk occurring in the 50–59 age-group, sub-hazard ratio 7.90 (5.38, 11.60), with risk decreasing steadily thereafter (Fig 3).

### Mortality

**Fig 4:**
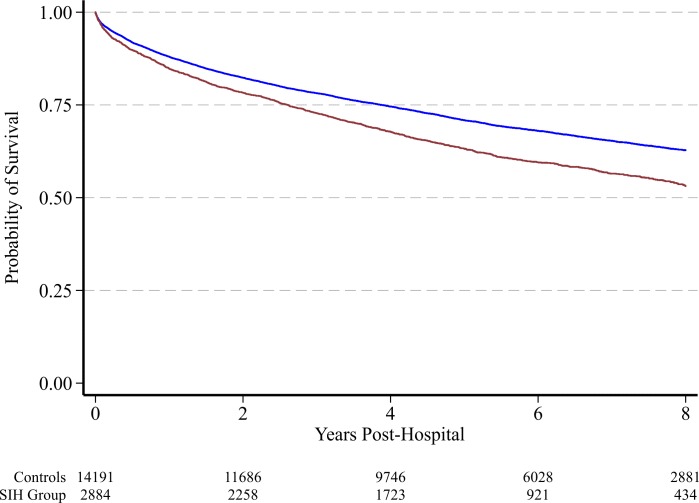
Kaplan-Meier survival curves for the control group (blue line) and stress induced hyperglycemia (red line) from hospital discharge to 8 years.

The 8 year mortality rate for survivors of critical illness without known diabetes was 34% (5,843 of 17,074). Unadjusted proportional hazards regression suggested an increased risk of death associated with stress induced hyperglycaemia (Fig 4); however, this signal was no longer present after adjusting for age and severity of illness (hazard ratio 1.04 (0.97, 1.11), *P =* 0.276).

## Discussion

The key finding of this study is that stress induced hyperglycemia appears to approximately double the risk of incident diabetes in survivors of critical illness. This is the largest study to assess the long term risk of incident diabetes in survivors of critical illness and includes all patients admitted to a public hospital ICU over a prolonged observational period.

The key finding is biologically plausible and consistent with epidemiological studies reporting an association between hyperglycemia during hospitalization and subsequent diabetes [16, 18, 20, 21]. The most externally valid of these, a retrospective cohort study of over 86,000 admissions to emergency departments in Scotland, reported that patients who are hyperglycemic (blood glucose > 11 mmol/L (200 mg/dL)) had a three-year risk of developing diabetes of 10%, compared to 2.3% in all patients admitted to the emergency department [16]. However, only 1,853 (2%) patients were admitted to an ICU with only 37 (0.04%) of these developing diabetes [16].

There are limited data relating to the relationship between stress induced hyperglycemia and the development of type 2 diabetes in survivors of critical illness [19, 22, 23]. In a prospective observational study from a single ICU, Gornik and colleagues performed annual oral glucose tolerance tests for 5 years in 582 survivors of critical illness, stratifying patients into normoglycemia and stress induced hyperglycemia based on peak inpatient blood glucose [19]. Patients with stress induced hyperglycemia (peak blood glucose > 7.8 mmol/l (140.4 mg/dL) had a five-fold increased risk of incident type 2 diabetes [19]. The external validity of these results is diminished by the lack of severity of illness data and the relatively high proportion of patients receiving total parenteral nutrition (32%) [19]. In a similar, single-centre study Van Ackerbock and colleagues performed an oral glucose tolerance test 8 months post ICU discharge in 338 survivors of critical illness [22]. Hyperglycemia (peak blood glucose > 7.8 mmol/L (140.4 mg/dL)) did not identify patients at risk of incident diabetes, possibly reflecting the short time period for diagnosis of incident diabetes and the relatively small sample size.

For this study, given that fasting status was unknown, stress induced hyperglycemia was defined using the American Diabetes Association Diabetes in Hospitals Writing Committee Guidelines, i.e. random blood glucose ≥ 11.1 mmol/L (200 mg/dL) [24]. This threshold has also been proposed as the cut-off at which screening programs may be beneficial [16]. The risk of incident diabetes varied according to age, with the greatest risk demonstrated in patients aged 50–59, and in this sub-group stress induced hyperglycemia was associated with a seven-fold increased risk of incident diabetes. This is likely to be important because for any diabetes screening program to be cost-effective they must identify younger populations who have the greatest capacity to benefit early intervention [25]. Accordingly, these data provide a persuasive rationale for the evaluation of screening programs in a relatively young population of ICU survivors.

This study has several important limitations. New registration with the NDSS was used as a surrogate marker for incident diabetes. While the true incidence of diabetes is likely higher than reported in this study, there is no reason to expect bias in NDSS registration between groups, and so NDSS registration is likely to be a valid surrogate.

Peak blood glucose in the first 24 hours was utilised as the sole metric for classifying stress induced hyperglycemia, missing those patients in whom hyperglycemia was delayed. While this may have resulted in some patient misclassification, it is reassuring that blood glucose concentrations within the first 24 hours are predictive of glycaemic control throughout ICU admission [26]. A further limitation was our inability to quantify whether risk was increased following sustained duration of stress induced hyperglycemia.

Feeding status was not available and patients with fasting blood glucose 7.0–11.0 mmol/L (126–198 mg/dL) [24] may have been missed. This is supported by the sharp rise in risk at a blood glucose ≥ 7.8 mmol/L (141 mg/dL), likely reflecting the population of fasting patients with unrecognised SIH and underestimation of the true hazard rate. Furthermore, insulin infusion protocols differed between units, which may have modified peak glycaemia. However, while there was a statistically significant difference in mean peak blood glucose between units, this was only 0.6 mmol/L (10.8 mg/dL) and unlikely to be of substantive clinical relevance or to have biased hazard estimates and was included in the multivariate model. Missing blood glucose or demographic data resulted in approximately 10% of patient separations being excluded from potential matching; however, this exclusion rate is comparable to previous cohort studies using blood glucose data from the ANZICS-APD and most frequently occurs with brief admissions where no blood tests are recorded [27]. There were no data available on body mass index (BMI) or other known risk factors for the development of diabetes such as alcohol consumption or family history of impaired glucose tolerance. It is somewhat reassuring however, that a prospective observational study in a similar cohort reported that once patients with undiagnosed diabetes are excluded increasing BMI does not identify a group of patients and increased risk of stress induced hyperglycemia [2]. Notwithstanding limitations inherent to epidemiological studies, it should be recognized that unmeasured factors may have confounded risk estimates. Future prospective studies that assess incident diabetes in survivors of critical illness and account for all potential confounders are therefore needed.

As glycated hemoglobin (HbA1c) is not routinely measured on ICU admission, the proportion of patients classified as having stress induced hyperglycemia who actually had prevalent but unrecognised type 2 diabetes is unknown. Previous epidemiological studies report the prevalence of unknown diabetes to be between 5 and 10% of patients admitted to ICU [2, 28]. To limit the likelihood of unknown diabetes being a major confounder prevalent diabetes was identified by a thorough process including verifying across two separate databases (using ICD-10 codes and NDSS registration) and excluding patients with a blood glucose > 20 mmol/L (360 mg/dL), or who registered with the NDSS within 30 days of hospital discharge, were excluded [16]. Moreover, in a recent study from one of the four participating ICUs 5.5% of admitted patients had previously unrecognised diabetes (i.e. HbA1c ≥ 6.5% (47.5 mmol/mol)) [2], which suggests that the rate of undiagnosed diabetes appears to be relatively infrequent in South Australia. Nonetheless, the potential that undiagnosed diabetes may have biased hazard estimates cannot be excluded.

### Clinical implications

In the U.S. alone annual admissions to intensive care units total more than 5.7 million [29]. Epidemiological data indicate that stress induced hyperglycaemia occurs frequently [2]. Accordingly, there are a large number of patients who survive ICU with stress hyperglycaemia who may benefit from earlier detection of incident diabetes. There are major benefits in identifying individuals at risk of incident diabetes, including prompt diagnosis facilitating earlier treatment thereby reducing complication rates [30, 31], with current guidelines in many regions recommend screening high-risk individuals [32]. Studies are now warranted to determine the efficacy of such screening programs for patients with stress induced hyperglycemia who survivor critical illness.

## Conclusions

Acute hyperglycemia during critical illness identifies patients at substantially greater risk of incident diabetes following hospital discharge. The risk of incident diabetes appears to be greatest in middle-aged patients, which may have implications for screening of this population.

## Supporting Information

S1 Fig Peak blood glucose by participating unit. (TIF)

S2 Fig Registration with the NDSS by peak blood glucose. Log-Odds (95% CI) for registration with the NDSS plotted against equally sized group centre values (x10) for peak blood glucose level by univariate logistic regression.
(TIF)

S1 Table Blood glucose concentration at which insulin was commenced by ICU Site. (DOCX)
