# Supplementary figures and images for: Stress Induced Hyperglycemia and the Subsequent Risk of Type 2 Diabetes in Survivors of Critical Illness
Source: PLoS One. 2016 Nov 8;11(11):e0165923. doi: 10.1371/journal.pone.0165923 (PMC5100960; doi:10.1371/journal.pone.0165923)

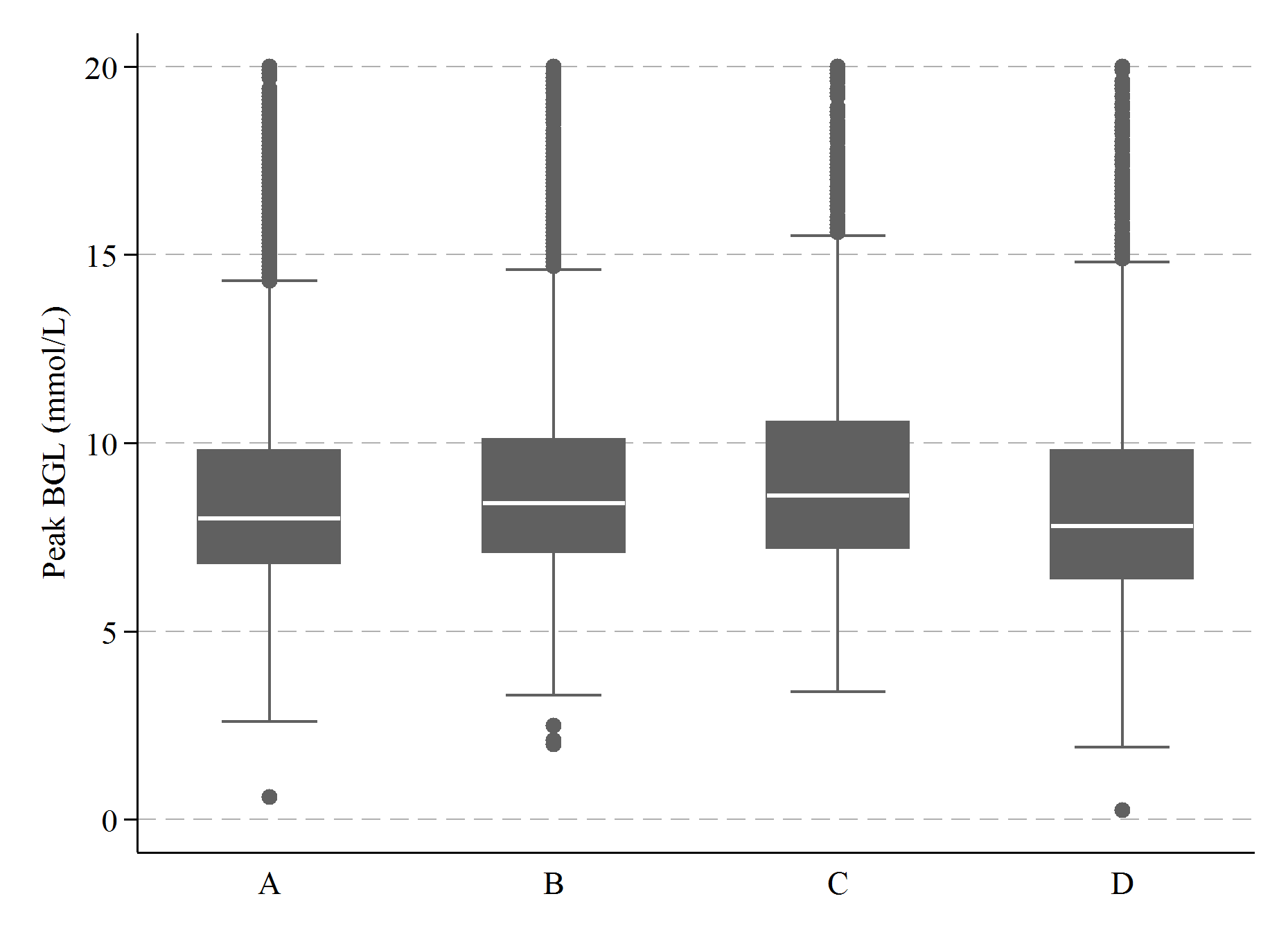

Supplement: S1 Fig — (TIF) [file pone.0165923.s001.tif]

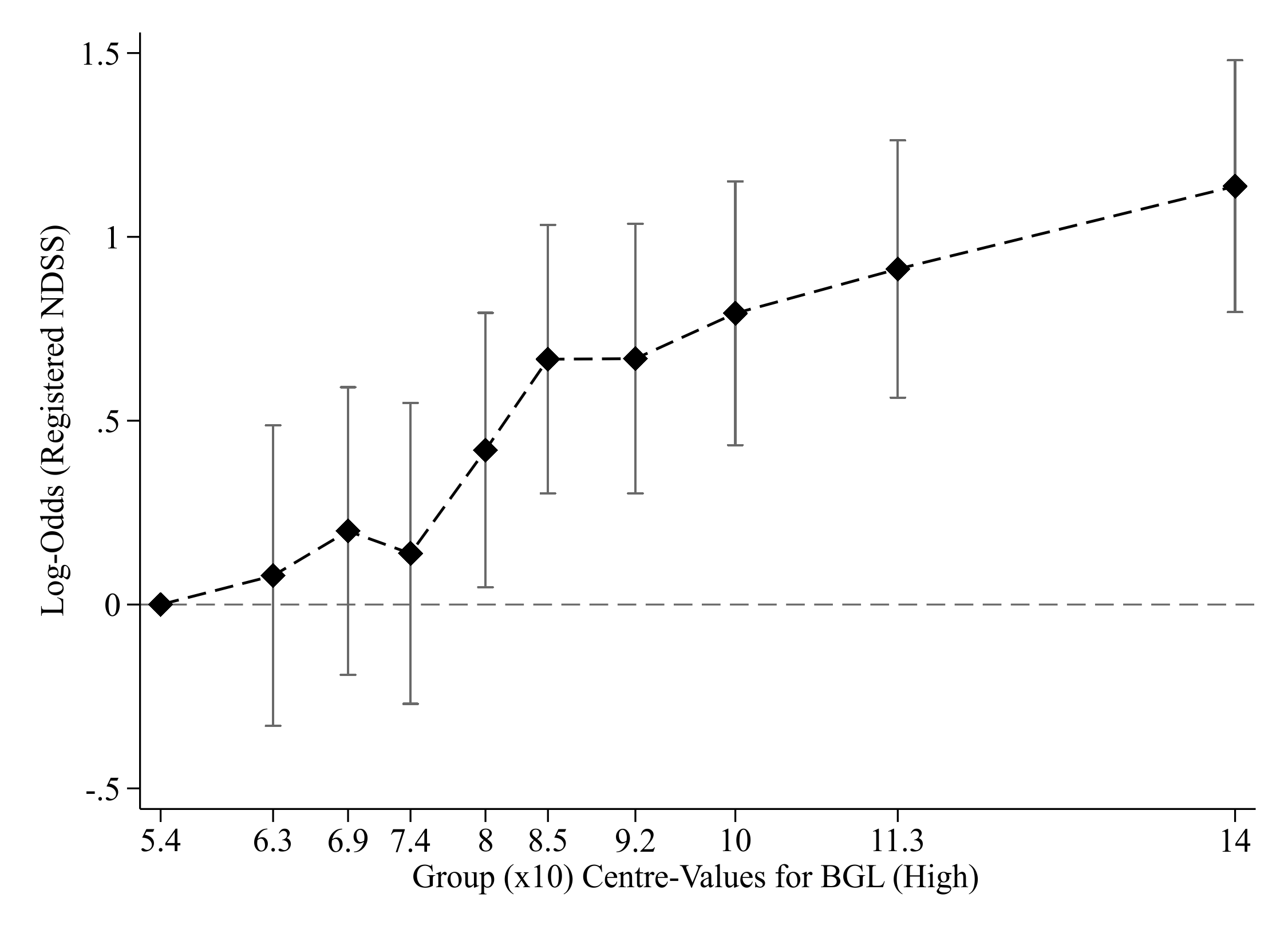

Supplement: S2 Fig — Log-Odds (95% CI) for registration with the NDSS plotted against equally sized group centre values (x10) for peak blood glucose level by univariate logistic regression. (TIF) [file pone.0165923.s002.tif]
