# Supplementary material for: Stress Induced Hyperglycemia and the Subsequent Risk of Type 2 Diabetes in Survivors of Critical Illness
Source: PLoS One. 2016 Nov 8;11(11):e0165923. doi: 10.1371/journal.pone.0165923 (PMC5100960; doi:10.1371/journal.pone.0165923)
Supplement: S1 Table — (DOCX) [file pone.0165923.s003.docx]

**Supplementary Table 1. Blood glucose concentration at which insulin was commenced by ICU Site**

| ICU Hospital Site | Calendar Period | Target BGL (mmol/l) |
| --- | --- | --- |
| A | January 2004 – December 2009 | ≥ 8.0 |
|  | January 2010 – December 2011 | ≥ 10.0 |
| B | January 2004 – December 2011 | ≥ 8.0 |
| C | January 2004 – November 2008 | ≥ 8.0 |
|  | November 2008 – December 2011 | ≥ 10.0 |
| D | January 2004 – April 2009 | ≥ 8.0 |
|  | April 2009 – December 2011 | ≥ 10.0 |
